# Comparative evaluation of BacT/ALERT VIRTUO and BACTEC FX400 blood culture systems for the detection of bloodstream infections

**DOI:** 10.1128/spectrum.01850-24

**Published:** 2024-11-29

**Authors:** Yurong Qin, Yiwen Liao, Jingfang Zhou, Weijiang Liu, Huimin Chen, Xiaoli Chen, Weisha Wang, Ni Zhang, Yunhu Zhao, Liang Wang, Bing Gu, Suling Liu

**Affiliations:** 1 Guangdong Cardiovascular Institute, Guangdong Provincial People’s Hospital, Guangdong Academy of Medical Sciences, Guangzhou, Guangdong, China; 2 Department of Laboratory Medicine, Guangdong Provincial People‘s Hospital (Guangdong Academy of Medical Sciences), Southern Medical University, Guangzhou, Guangdong, China; Children's National Hospital, George Washington University, Washington, DC, USA

**Keywords:** bloodstream infections (BSI), blood culture systems, diagnostic accuracy, pathogen detection, comparative analysis, intensive care units (ICU)

## Abstract

Bloodstream infections (BSI) pose significant threats to patient health, necessitating timely and accurate diagnostics to reduce mortality and morbidity. This study aimed to evaluate the clinical performance of the BacT/ALERT VIRTUO blood culture system with FANPlus bottles compared to the BACTEC FX400 system in detecting bloodstream pathogens. A total of 1,772 blood specimens were collected from various hospital wards. Specimen selection criteria were based on clinical suspicion of bloodstream infections, ensuring the inclusion of relevant and representative patient samples. These blood samples, collected from the same suspected sepsis patients, were cultured in parallel using both the BacT/ALERT VIRTUO and BACTEC FX400 systems. The diagnostic efficiency of both systems, including detection rates, time to detection (TTD), and sensitivity across different bacterial species, was assessed. In various application scenarios, the VIRTUO system demonstrates a higher positive detection rate, whether in the intensive care unit (ICU) (8.5% vs 6.4%, *P* = 0.028) or in general wards. Additionally, for different types of bacteria, the TVIRTUO system exhibits superior detection rates for anaerobic bacteria (5.9% vs 3.2%, *P* < 0.001) and aerobic bacteria (9.1% vs 7.2%, *P* = 0.043). Furthermore, it boasts a shorter median TTD of 14 hours compared to 16 hours, and a higher sensitivity for Gram-positive bacteria (2.8% vs 1.6%, *P* < 0.001). These findings emphasize VIRTUO’s effectiveness in enhancing diagnostic accuracy, achieving faster time to detection, and expanding the spectrum of detected organisms, thereby facilitating the quicker initiation of appropriate therapies, supporting precise clinical decision-making, and ultimately improving patient outcomes.

**IMPORTANCE:**

Our study conducted a critical evaluation of advanced blood culture technologies for managing bloodstream infections (BSI). A distinctive strength of our research is the large sample size and the concurrent testing of the same patients with two systems, a methodology rarely achieved in other studies. BSIs present severe health threats, necessitating prompt and accurate diagnostics to mitigate morbidity and mortality. The BacT/ALERT VIRTUO system, in comparison to the BACTEC FX400 system, demonstrated superior detection capabilities, emphasizing the critical role of advanced diagnostics in clinical settings.

## INTRODUCTION

Bloodstream infections (BSI) represent severe clinical conditions resulting from the presence of pathogens or their toxins in the bloodstream, leading to systemic inflammatory response syndrome, a widespread inflammatory reaction that can result in organ dysfunction, posing significant threats to patient health. If not diagnosed and treated promptly, BSIs can result in severe complications such as septic shock, multi-organ failure, and significantly increased mortality rates, underscoring the critical need for timely and accurate diagnosis (1–3). The timely and accurate diagnosis and treatment of BSI are essential, as they can substantially reduce mortality rates, minimize complications, and shorten hospital stays (4–6). However, despite its critical role, blood culture methods face significant challenges that impede optimal patient management. In this regard, blood culture remains the gold standard for detecting microorganisms in the bloodstream, playing a pivotal role in managing BSI. However, current blood culture systems, including the BACTEC FX400, are limited by delayed detection times and suboptimal sensitivity, particularly in identifying fastidious organisms—those with complex nutritional or environmental requirements that make them challenging to detect through conventional culture methods. This study evaluates whether the BacT/ALERT VIRTUO system, with its advanced features, can address these limitations.

In recent years, new blood culture systems have been developed to improve the time to detection (TTD) and sensitivity of pathogen recovery. For instance, the Mindray TDR-X060 system has shown promising results, with detection times for yeasts being significantly shorter than those of the BacT/ALERT 3D system at certain bacterial concentrations (7). Moreover, the VIRTUO system, which is evaluated in this study, has demonstrated faster TTD for many clinically relevant species when compared to both the BACTEC and BacT/ALERT 3D systems, especially for pathogens like *Salmonella* spp. and *Streptococcus agalactiae* (8). These advancements reflect the ongoing technological improvements in blood culture systems, aiming to reduce diagnostic delays and enhance the detection of bloodstream pathogens.

Blood cultures enable the isolation and identification of causative pathogens. They also facilitate antibiotic susceptibility testing, which determines a pathogen’s resistance or sensitivity to antibiotics, providing critical information for clinicians to select the most effective antimicrobial therapy (9). Moreover, blood cultures facilitate the monitoring of therapeutic efficacy, ensuring complete eradication of the pathogens (10, 11). The role of blood culture is indispensable throughout the diagnostic, therapeutic, and monitoring phases of BSI, as it aids in identifying a wide range of pathogens, including bacteria and fungi, and provides essential epidemiological data to guide public health interventions. Thus, the application of blood culture is paramount in enhancing the diagnosis and management of BSI (12–14).

Despite its crucial role, blood culture faces numerous challenges and key issues in current clinical and scientific practice. First, the sensitivity and specificity of blood cultures can be suboptimal, leading to false-positive and false-negative results (15, 16). False-positive results, often caused by contamination during sample collection, complicate the interpretation of culture findings and may lead to unnecessary antimicrobial treatments, increasing the risk of antimicrobial resistance and unnecessary healthcare costs. Conversely, false-negative results occur when the bacterial load is too low or the causative organism is fastidious and does not grow well in standard culture media, thereby delaying an accurate diagnosis and the initiation of appropriate therapy. Second, the prolonged time required to obtain blood culture results, typically ranging from 24 to 48 hours for preliminary results and several days for final identification and susceptibility testing, can delay the initiation of targeted antimicrobial therapy. These challenges emphasize the need for advanced blood culture systems that can enhance both sensitivity and detection speed. Additionally, inadequate blood sample volumes and infrequent sampling can reduce the likelihood of pathogen detection (17, 18). The issue of antibiotic pretreatment is also significant, as patients who have received empirical antibiotic therapy before blood culture sampling may have reduced bacterial loads, leading to false-negative results. Furthermore, the emergence of novel pathogens and antimicrobial resistance presents major challenges, as conventional blood culture methods may not always detect these pathogens or provide timely susceptibility data (19, 20). Addressing these challenges requires advances in blood culture techniques, including the development of improved culture media and growth conditions, the integration of molecular diagnostic technologies such as the polymerase chain reaction and next-generation sequencing, and the implementation of automated blood culture systems and rapid diagnostic tests (21, 22). Additionally, optimizing blood sample collection protocols and enhancing antimicrobial resistance surveillance are critical for improving the diagnostic accuracy and clinical utility of blood cultures.

Our study highlights the importance of comparing the clinical performance of the BacT/ALERT VIRTUO and BACTEC FX400 systems, particularly in relation to detection speed, sensitivity, and pathogen spectrum. Optimizing such diagnostic technologies is critical for improving patient outcomes by enabling timely and accurate antimicrobial therapy, especially in the ongoing fight against antimicrobial resistance. The BacT/ALERT VIRTUO system demonstrated enhanced growth conditions, faster time to detection, and improved sensitivity in detecting a broader range of pathogens, including anaerobic and fastidious organisms, which were key factors in its selection for this study.

## MATERIALS AND METHODS

### Inclusion criteria

Patients, aged 16 years and above with an approximately equal gender distribution, were selected from clinical internal medicine and intensive care unit (ICU) wards, including Hematology, Gynecology, Infectious Diseases ward, Respiratory Department (Ward 1 and Ward 2), Intensive Care Unit 1, Intensive Care Unit 2, Geriatric Intensive Care Unit, and Cardiac Care Unit. For patients suspected of having sepsis in these wards (based on the following criteria: (i) fever [≥38°C] or hypothermia [≤36°C]; (ii) chills; (iii) leukocytosis [>10×10^9/L] with a left shift or leukopenia [<3×10^9/L]; (iv) skin or mucosal bleeding; (v) coma; (vi) multi-organ failure; and (vii) hypotension…), blood was drawn from two puncture sites, with each site providing 8–10 mL of blood, totaling two sets of blood culture samples. When the volume of blood in each bottle is less than 5 mL, false negatives or delayed microbial growth may occur. When the volume exceeds 10 mL per bottle, false positives can result from the large amount of carbon dioxide produced by white blood cells. Patients with improper blood collection techniques and those with severe anemia or hypovolemia, conditions that may lead to significant sampling errors due to reduced blood volume, were excluded from the study. To prevent contamination, venipuncture sites were wiped with 75% ethanol for over 30 seconds; iodine tincture was applied for 30 seconds, disinfecting in a circular motion from the puncture site outward until the disinfected area had a diameter of at least 3 cm; 75% ethanol was then used to remove the iodine, and the area was allowed to dry. One set of blood culture samples was injected into the VIRTUO system (FA plus & FN plus bottles) (BacT/ALERT VIRTUO, bioMérieux, France), and the other set was injected into the BACTEC FX instrument 400 (resin aerobic bottle and lysing anaerobic bottle) (Becton Dickinson, Cockeysville, USA). The turnaround time from blood collection to loading into the system should be ≤2 hours. If it exceeds 2 hours, it may affect the detection of positive results, leading to missed diagnoses.

### Blood culture process

#### Primary report

Positive blood culture bottles were immediately subcultured on agar plates, including blood agar, Sabouraud’s agar, chocolate agar, and MacConkey agar. Smears were stained using Gram and Wright staining methods, and the staining results constituted the “primary blood culture report,” which was issued as a “critical value” to the clinical team within 30 minutes.

#### Secondary report

Subcultured plates were incubated at 35°C for 6–8 hours, followed by species identification using the VITEK MS mass spectrometer (Vitek MS, bioMérieux, France). The identification results constituted the “secondary blood culture report,” and after review and confirmation, the report was issued to the clinical team. Concurrently, based on the identification results, an *in vitro* antimicrobial susceptibility test was conducted and the final report was issued. The final report included the identification of bacterial species and antimicrobial susceptibility results based on the species identification. If no positive results were observed after 120 hours of blood culture, a negative report was issued, stating: “No aerobic bacterial growth, no anaerobic bacterial growth, and no fungal growth after 5 days of incubation.”

### Bacterial identification method

All positive bacterial strains were identified using the VITEK MS mass spectrometer (Vitek MS, bioMérieux, France), as previously reported (23, 24). To ensure accuracy, the VITEK MS system was calibrated regularly following manufacturer protocols and internal quality control measures were implemented throughout the study. The identification results were cross-validated with conventional biochemical methods for selected strains, confirming the reliability of the mass spectrometry identification process. However, limitations of the VITEK MS system include potential difficulties in identifying rare or novel pathogens, which may not be well-represented in the system’s database. In such cases, additional confirmatory methods, such as molecular diagnostics or extended culture techniques, may be required.

### *In vitro* antimicrobial susceptibility testing

Based on the species identification results from the VITEK MS mass spectrometer, appropriate antimicrobial susceptibility cards were selected for automated *in vitro* susceptibility testing using the VITEK2 Compact (bioMérieux, France) microbial analysis system. For some strains, supplementary testing, including the Kirby-Bauer method and broth micro-dilution method, was conducted. These additional methods were applied when discordant results were observed between the automated VITEK2 system and clinical expectations, or when the bacterial species were known to have variable susceptibility profiles that required further validation. The final antimicrobial susceptibility results were determined by integrating the results from these three methods (25). When discrepancies were identified between methods, priority was given to the broth micro-dilution method, which is considered the gold standard. In cases of conflicting data, expert clinical judgment and reference to established guidelines, such as those from CLSI or EUCAST, were used to reconcile differences and produce the final report.

### Statistical analysis

The data analyses for this study were primarily conducted using Excel 2016 and SPSS version 26. The statistical methods employed included Pearson’s Chi-squared test for comparing categorical variables and the Wilcoxon rank sum test for comparing non-normally distributed continuous variables between different groups. These methods were selected to assess the significance of observed differences across various data points. In this study, missing data were handled using multiple imputations. This method was applied to generate complete data sets by replacing missing values multiple times, ensuring the integrity of the analysis and the reliability of the results. The handling of missing data did not have a significant impact on the outcomes. Due to potential system synchronization issues within the hospital, instances may occur where the VIRTUO system reports a positive blood culture result, but no bacterial growth is ultimately detected. Such cases were classified as false positives and were excluded from the data set to ensure data integrity.

## RESULTS

### BacT/ALERT VIRTUO system shows higher positive detection rates across various wards

In general, different detection systems exhibit varying positive detection rates across hospital departments. Table 1 shows that, for the FX400 system, the positive detection rate in the ICU is significantly higher than that in general wards (6.4% vs 4.2%, *P* = 0.002). Similarly, for the BacT/ALERT VIRTUO system, the positive detection rate in the ICU is also significantly higher than in general wards (8.5% vs 6.7%, *P* = 0.043). Across different departments, there is also a significant difference in positive detection rates between the BacT/ALERT VIRTUO and BACTEC FX400 systems. As shown in Table 2, in the ICU, among 1,628 blood samples, the FX400 system detected 105 positive cases (detection rate of 6.4%), which is significantly lower than the 138 cases detected by the VIRTUO system (detection rate of 8.5%, *P* = 0.028). In 1,916 samples from general wards, the positive detection rate of the VIRTUO system was also significantly higher (6.7%, 128 cases) compared to the FX400 system (4.2%, 80 cases, *P* < 0.001). Overall, these findings indicate that both BacT/ALERT VIRTUO and BACTEC FX400 systems have higher positive detection rates in the ICU, with the BacT/ALERT VIRTUO system showing superior positive detection efficiency compared to the BACTEC FX400 system, in both ICU and general ward settings.

**TABLE 1:** Comparison of the positive detection rate between VIRTUO and FX400 systems

Characteristic	FX400, *N* = 3,544			VIRTUO, *N* = 3,544		
	Intensive care unit, *N* = 1,628^*a*^	General wards, *N* = 1,916^*a*^	*P*-value^*b*^	Intensive care unit, *N* = 1,628^*a*^	General wards, *N* = 1,916^*a*^	*P*-value^*b*^
Analyze the results			0.002			0.043
Positive	105 (6.4%)	80 (4.2%)		138 (8.5%)	128 (6.7%)	
Negative	1,523 (93.6%)	1,838 (95.8%)		1,490 (91.5%)	1,788 (93.3%)	

^*a*^ n(%).

^*b*^ Pearson's Chi-squared test.

**TABLE 2:** Comparison of the positive detection rate in different departments

Characteristic	Intensive care unit, *N* = 3,256			General wards, *N* = 3,832		
	FX400, *N* = 1,628^*a*^	VIRTUO, *N* = 1,628^*a*^	*P*-value^*b*^	FX400, *N* = 1,916^*a*^	VIRTUO, *N* = 1,916^*a*^	*P*-value^*b*^
Analyze the results			0.028			<0.001
Positive	105 (6.4%)	138 (8.5%)		80 (4.2%)	128 (6.7%)	
Negative	1,523 (93.6%)	1,490 (91.5%)		1,836 (95.8%)	1,788 (93.3%)	

^*a*^ n(%).

^*b*^ Pearson's Chi-squared test.

### BacT/ALERT VIRTUO system demonstrates a higher positive detection rate for both anaerobic and aerobic bacteria compared to the BACTEC FX400 system

We conducted an analysis of the positive detection rates for different types of blood vials—specifically, anaerobic bottles and aerobic cylinders—using the FX400 and VIRTUO detection systems, as shown in Table 3.

**TABLE 3:** Comparison of positive detection rates among blood vial types of different detection systems

Variable	Anaerobic bottles, *N* = 3,544			Aerobic cylinders, *N* = 3,544		
	FX400, *N* = 1,772^*a*^	VIRTUO, *N* = 1,772^*a*^	*P*-value^*b*^	FX400, *N* = 1,772^*a*^	VIRTUO, *N* = 1,772^*a*^	*P*-value^*b*^
Analyze the results			<0.001			0.043
Positive	57 (3.2%)	105 (5.9%)		128 (7.2%)	161 (9.1%)	
Negative	1,715 (96.8%)	1,667 (94.1%)		1,644 (92.8%)	1,611 (90.9%)	

^*a*^ n(%).

^*b*^ Pearson's Chi-squared test.

Among 3,544 anaerobic bottle specimens, the FX400 system identified 57 positive cases (3.2%), while the VIRTUO system detected 105 positive cases (5.9%). This difference in detection rates is statistically significant (*P* < 0.001), indicating that the VIRTUO system has a higher positive detection rate for anaerobic bacteria compared to the FX400 system. In the detection of 3,544 aerobic bottle specimens, the FX400 system identified 128 positive cases (7.2%), whereas the VIRTUO system identified 161 positive cases (9.1%). This difference is also statistically significant (*P* = 0.043), demonstrating that the VIRTUO system has a higher positive detection rate for aerobic bacteria compared to the FX400 system. Overall, these findings suggest that the VIRTUO system shows superior positive detection rates over the FX400 system for both anaerobic and aerobic bacteria.

### BacT/ALERT VIRTUO system demonstrates faster TTD compared to BACTEC FX400

We conducted an analysis of the TTD between the FX400 and VIRTUO detection systems, as shown in Table 4.

**TABLE 4:** Comparison of positive detection time of different detection systems

Variable	FX400, *N* = 3,544^*a*^	VIRTUO, *N* = 3,544^*a*^	*P*-value^*b*^
**Time**	16(12,27)	14(9,21)	**<0.001^*c*^**

^*a*^ Median (IQR).

^*b*^ Wilcoxonrank sum test.

^*c*^ The bold values represent results with p-values significantly smaller than 0.001, indicating very strong statistical significance.

**Fig 1:**
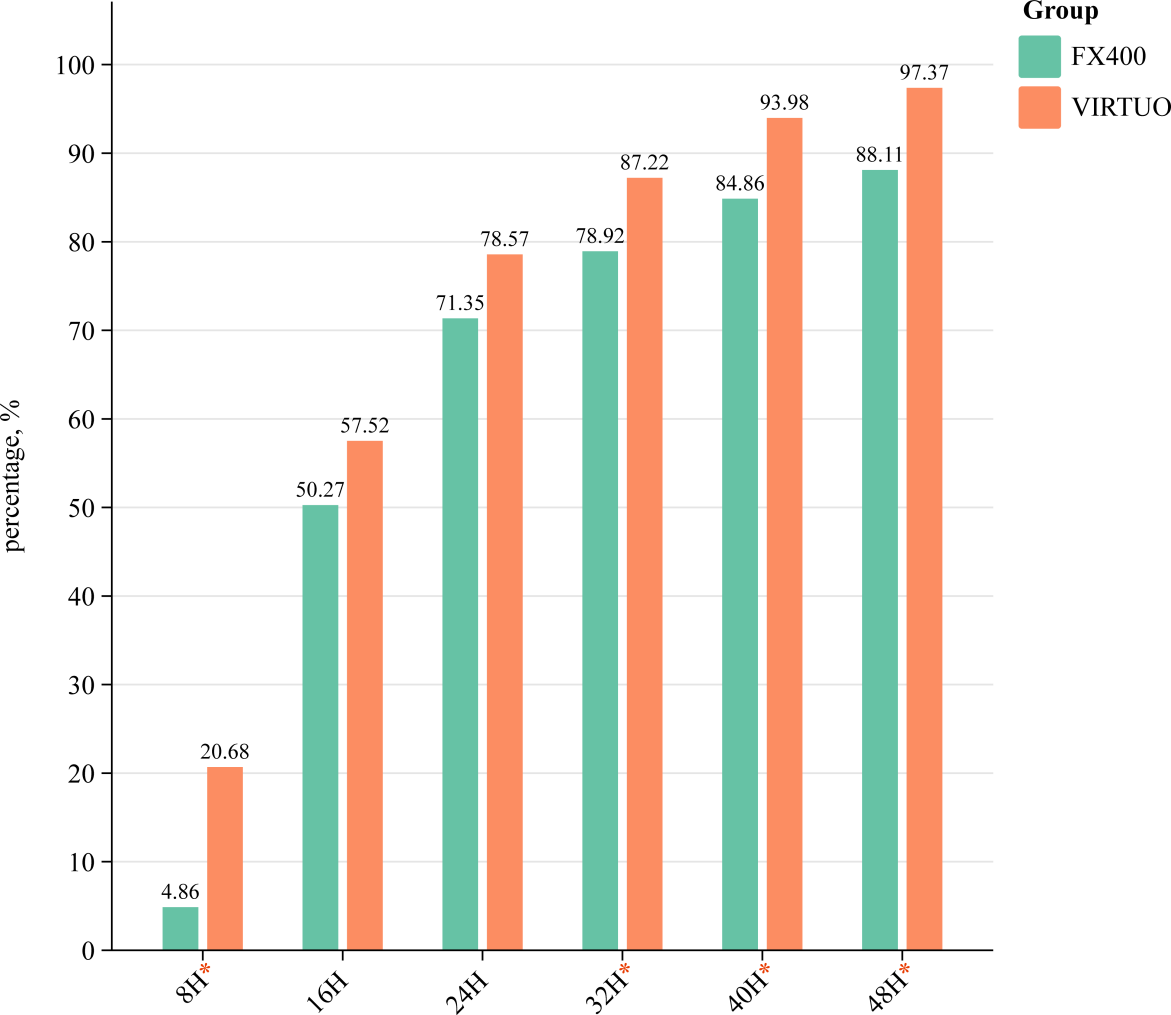
Cumulative TTD distribution for BacT/ALERT VIRTUO and BACTEC FX400 systems. This figure illustrates the cumulative percentages of positive detection rates for BacT/ALERT VIRTUO and BACTEC FX400 over various time intervals. VIRTUO demonstrates consistently higher detection rates at each interval, starting from 0 to 8 hours and continuing through 48 hours. These results indicate that VIRTUO provides faster diagnostic feedback compared to FX400, particularly in the critical early detection windows, which can be instrumental in timely clinical interventions. An asterisk (*) denotes significant differences; see the (Table S1) for statistical details.

The study results show that the median TTD for positive samples in the FX400 system is 16 hours (IQR: 12–27). In contrast, the VIRTUO system has a shorter median TTD of 14 hours (IQR: 9–21), indicating a faster detection rate compared to FX400 (*P* = 0.001) (Table 4). The cumulative TTD distribution shown in Fig. 1 demonstrates that the BacT/ALERT VIRTUO system achieves quicker and more efficient detection than the BACTEC FX400. Within the first 8 hours, VIRTUO detected 20.68% of positive samples, significantly higher than the 4.86% detected by FX400 (*P* < 0.001). After 16 and 24 hours, the positive detection rates of VIRTUO were 57.52% and 78.57%, respectively, both higher than FX400’s rates of 50.27% and 71.35%, though these differences were not statistically significant (*P* < 0.05). After 32 hours, VIRTUO’s detection rate reached 87.22%, while FX400 reached 78.92%. At 40 hours, VIRTUO’s detection rate was 93.98%, compared to FX400’s 84.86%, and by 48 hours, VIRTUO achieved a positive detection rate of 97.37%, significantly higher than FX400’s 88.11% (all *P* < 0.05) (Fig. 1). These results indicate that VIRTUO consistently demonstrates a higher cumulative detection rate and stronger positive detection capability across different detection times.

### BacT/ALERT VIRTUO system exhibits higher positive detection rates across different bacterial species compared to BACTEC FX400

Compared to the BACTEC FX400 system, the BacT/ALERT VIRTUO system demonstrates an advantage in positive detection rates across various pathogen types (Table 5). Results from the study show that, out of 3,544 blood specimens, the VIRTUO system identified 266 positive cases (7.5%), while the FX400 system detected 185 positive cases (5.2%), a statistically significant difference (*P* < 0.001), indicating that VIRTUO has a notably higher overall positive detection rate. For Gram-positive bacteria, the VIRTUO system detected 101 positive cases (2.8%) compared to 55 cases (1.6%) in the FX400 system, showing significantly higher sensitivity of the VIRTUO system for Gram-positive pathogens. Although the BacT/ALERT VIRTUO system also had higher detection rates for fungi (0.8% vs 0.6%), Gram-negative bacteria (3.4% vs 2.8%), and mixed pathogens (0.5% vs 0.3%) than FX400, these differences did not reach statistical significance (*P* > 0.05), possibly due to the limited sample size. While these differences are not statistically significant, the VIRTUO system consistently showed higher detection rates across all pathogen types and particularly high sensitivity for Gram-positive bacteria, suggesting it may offer broader coverage in identifying bloodstream infections compared to the FX400 system. Further research with a larger data set is needed to confirm these observations.

**TABLE 5:** Comparison of detection rates for different organism classes between BacT/ALERT VIRTUO and BACTEC FX400 systems

Characteristic		FX400, *N* = 3,544^*a*^	VIRTUO, *N* = 3,544^*a*^	*P*-value^*b*^
Total	Positive	185 (5.2%)	266 (7.5%)	<0.001
	Negative	3,359 (94.8%)	3,278 (92.5%)	
Type of culture				
Fungal	Positive	20 (0.6%)	27 (0.8%)	0.306
	Negative	3,524 (99.4%)	3,517 (99.6%)	
Gram-negative bacteria	Positive	99 (2.8%)	121 (3.4%)	0.132
	Negative	3,445 (97.2%)	3,423 (96.6%)	
Gram-positive bacteria	Positive	55 (1.6%)	101 (2.8%)	<0.001
	Negative	3,489 (98.4%)	3,443 (97.2%)	
Mixed bacteria	Positive	12 (0.3%)	18 (0.5%)	0.272
	Negative	3,532 (99.7%)	3,526 (99.5%)	

^*a*^ n(%).

^*b*^ Pearson's Chi-squared test.

Table 6 provides a comparison of detection outcomes between the BacT/ALERT VIRTUO and BACTEC FX400 blood culture systems for a variety of bacterial and yeast species. The results are presented in three columns: the number of patient cases detected as positive by both systems, the number of positive cases detected exclusively by the VIRTUO system, and the number detected exclusively by the FX400 system. Overall, VIRTUO demonstrated a higher detection rate, identifying 44.86% of cases exclusively, while FX400 detected only 16.46% exclusively. When categorized by Gram-positive bacteria, Gram-negative bacteria, and yeasts, VIRTUO consistently showed superior detection capabilities. In particular, VIRTUO exhibited enhanced sensitivity for detecting certain fastidious organisms, such as Bacteroides fragilis, Prevotella bivia, and Fusobacterium nucleatum, which are challenging to culture and require specific growth conditions. These findings indicate that the VIRTUO system provides broader diagnostic coverage and improved detection sensitivity, especially for fastidious pathogens.

**TABLE 6:** Comparison of detection outcomes for BacT/ALERT VIRTUO and BACTEC FX400 blood culture systems across bacterial and yeast species

Bacterial species	Both positive		VIRTUO positive only		FX400 positive only	
Gram-positive	28	30.43%	51	55.43%	13	14.13%
*Streptococcus constellatus*	1	100.00%				
*Staphylococcus capratus*			1	100.00%		
*Streptococcus pneumoniae*	2	100.00%				
*Staphylococcus epidermidis*	4	18.18%	14	63.64%	4	18.18%
*Enterococcus faecalis*	3	42.86%	2	28.57%	2	28.57%
*Staphylococcus aureus*	8	44.44%	8	44.44%	2	11.11%
*Staphylococcus capitis*	1	25.00%	2	50.00%	1	25.00%
*Micrococcus luteus*			1	100.00%		
*Staphylococcus hominis*	1	10.00%	8	80.00%	1	10.00%
*Staphylococcus haemolyticus*	3	50.00%	2	33.33%	1	16.67%
*Enterococcus faecium*	2	50.00%	1	25.00%	1	25.00%
*Bacillus cereus*			2	100.00%		
*Micrococcus luteus*			1	100.00%		
*Staphylococcus woerneri*			1	100.00%		
*Corynebacterium striatum*	2	50.00%	2	50.00%		
*Gram positive bacilli*			1	100.00%		
*Streptococcus mutans*	1	50.00%	1	50.00%		
*Propionibacterium acnes*			4	80.00%	1	20.00%
Gram-negative	54	46.61%	43	36.44%	21	17.79%
*Salmonella typhimurium*	1	100.00%				
*Enterobacter cloacae complex*	1	100.00%				
*Acinetobacter baumannii*	6	42.86%	6	42.86%	2	14.29%
*Escherichia coli*	15	42.86%	16	45.71%	4	11.43%
*Klebsiella pneumoniae*	13	52.00%	7	28.00%	5	20.00%
*Proteus mirabilis*	1	20.00%	2	40.00%	2	40.00%
*Steinotrophomonas maltophilia*	2	40.00%	2	40.00%	1	20.00%
*Pseudomonas aeruginosa*	10	76.92%	2	15.38%	1	7.69%
*Serratia marcescens*	1	100.00%				
*Burkholderia multiphaga*	1	33.33%	2	66.67%		
*Salmonella serotype Dublin*			1	100.00%		
*Salmonella group*	1	100.00%				
*Fusobacterium nucleatum*	1	100.00%				
*Prevotella polymorpha*	1	100.00%				
*Eikenella corrodens*					1	100.00%
*Bacteroides fragilis*			2	100.00%		
*Prevotella bivia*			1	100.00%		
*Pseudomonas putida*			2	100.00%		
*Cronobacter sakazakii*					1	100.00%
*Achromobacter xylosoxidans*					2	100.00%
*Sphingomonas paucimobilis*					1	100.00%
*Ralstonia insidiosa*					1	100.00%
Yeasts	12	36.36%	15	45.45%	6	18.18%
*Candida parapsilosis*	3	50.00%	2	33.33%	1	16.67%
*Talaromyces marneffei*	2	100.00%				
*Candida albicans*	5	35.71%	6	42.86%	3	21.43%
*Candida tropicalis*	2	28.57%	4	57.14%	1	14.29%
*Candida tenerife*			1	100.00%		
*Cryptococcus neoformans*			1	100.00%		
*Candida pseudopsilosis*			1	100.00%		
*Candida glabrata*					1	100.00%
Summary	94	38.68%	109	44.86%	40	16.46%

## DISCUSSION

The present study demonstrated that the BacT/ALERT VIRTUO system outperformed the BACTEC FX400 system in terms of overall positive detection rates, particularly in intensive care units. This higher detection rate was especially pronounced in ICUs, where timely and accurate detection of BSIs is crucial for patient outcomes. In clinical scenarios involving immunocompromised patients or those experiencing severe sepsis, a faster TTD can be particularly impactful (26–28). For instance, in our study, we observed a case involving a neutropenic patient who was highly susceptible to bloodstream infections. Rapid identification of pathogens enabled the prompt administration of targeted antimicrobial therapy, which was essential in preventing severe complications like septic shock or multi-organ failure. Such timely interventions can improve survival rates, shorten hospital stays, and reduce the overall cost of care.

The VIRTUO system performed particularly well in the ICU, largely due to the excellent adsorption capabilities of the FA PLUS aerobic and FN PLUS anaerobic bottles used in conjunction with VIRTUO for absorbing antimicrobial agents in the blood of ICU patients. Compared to other clinical departments, ICU patients are treated with a higher variety and intensity of high-level (special or restricted) antibiotics due to the critical condition and treatment complexity. For instance, carbapenem antibiotics (e.g., imipenem, meropenem) are frequently used to treat Gram-negative bloodstream infections, and daptomycin and vancomycin are commonly administered for Gram-positive bloodstream infections in ICU patients.

FA PLUS and FN PLUS bottles, as FDA-approved, have excellent adsorption performance for these antibiotics, achieving 100% absorption at peak blood concentrations. This eliminates the interference of antibiotics on blood culture positivity, optimizing the detection of pathogens in blood cultures. This enhanced capability of the VIRTUO system in both anaerobic and aerobic bottles contributes to its overall advantage in detecting a broader range of pathogens, including those that are more difficult to culture. The patented additives play a key role in overcoming the inhibitory effects of high-level antibiotics present in ICU patients’ blood, thereby ensuring more accurate detection of bloodstream infections. The detailed analysis of positive rates across different hospital wards provided granular insights into the performance of the BacT/ALERT VIRTUO and BACTEC FX400 systems, helping to identify specific areas where the VIRTUO system might be particularly beneficial. Such detailed departmental performance analysis is often overlooked in broader studies, yet it is crucial for tailoring diagnostic strategies to the specific needs of different hospital wards.

The superior detection capabilities of the VIRTUO system underscore its effectiveness in identifying a broader range of pathogens, which is critical for comprehensive clinical diagnoses (8, 29). In terms of pathogen detection, the BacT/ALERT VIRTUO system identified a greater number of bacterial species, including fungi and fastidious organisms. For instance, in aerobic bottles, the VIRTUO system detected 161 positive samples compared to 128 by the FX400 system. The difference is even more pronounced in anaerobic bottles, with the VIRTUO system detecting 105 positive samples against 57 by the FX400 system. In addition to its superior overall detection rates, the VIRTUO system also demonstrated significantly better performance in positive detection rates across different blood vial types. Its superior performance in detecting anaerobic organisms is particularly noteworthy. Anaerobic bottles can culture both facultative anaerobes and obligate anaerobes. The gas composition in anaerobic bottles is more conducive to the rapid detection of facultative anaerobes compared to aerobic bottles. Furthermore, the anaerobic bottles used in the VIRTUO system contain patented additives (which are absent in aerobic bottles). These additives covalently bind to certain antibiotics, such as carbapenems, allowing for faster and more effective neutralization of these antibiotics, which in turn improves the positive detection rate and shortens the time to detection.

TTD is another critical factor in managing BSI, as faster detection allows for earlier initiation of targeted therapies (30). It varies depending on the type of microorganism. Common Gram-negative bacteria typically report positive results within 24 hours, while Candida species take slightly longer, requiring approximately 30 hours for detection. Due to differences in pathogen growth characteristics, some organisms, such as filamentous fungi, may require 4–5 days for culture, and slow-growing bacteria like Brucella can take even longer, over 5 days. Additionally, for patients undergoing treatment with high doses of advanced antibiotics, the antimicrobial agents in their blood may inhibit pathogen growth, making it difficult to detect the pathogens in blood culture bottles. The BacT/ALERT VIRTUO system consistently showed faster detection times compared to the BACTEC FX400 system across various categories, including Gram-positive and Gram-negative bacteria, as well as aerobic and anaerobic bottles (31). For instance, the VIRTUO system had a median detection time of 14 hours (IQR: 9–21), compared to the FX400 system’s median detection time of 15 hours (IQR: 11–27) (*P* = 0.001). This early detection capability is particularly important in clinical settings, as it enables healthcare providers to quickly adjust treatment plans based on timely and accurate identification of pathogens, thereby improving patient outcomes. The study’s methodological rigor, including dual-site blood collection and culturing in different systems, minimized sample contamination and ensured accurate comparisons, making the findings robust and reliable. The ability to detect infections earlier allows for more prompt treatment interventions, which can be critical in patients with severe infections and can significantly impact patient prognosis (32, 33).

The performance difference between the BacT/ALERT VIRTUO and BACTEC FX400 systems may be attributed to several key factors. First, the VIRTUO system features a closed design, which prevents the need to open the device during loading and unloading, thereby maintaining a stable internal temperature. This minimizes the effect of temperature fluctuations on the positive detection curve. In contrast, the FX400 system uses an open-box design, where opening the box can lead to temperature changes that affect readings and introduce variability into the positive detection curve.

Additionally, the VIRTUO system’s positive detection algorithms offer several advantages. VIRTUO employs three major algorithms: Relative Area Under the Curve (RAUC) to enhance detection sensitivity, Read-to-Read (R2R) variation analysis to reduce detection time, and Early Incubation (EI) analysis to delay positive detection for slow-growing organisms.

There are also differences in the principles of detection. VIRTUO uses visible light for reading blood culture bottles, which provides stable measurements. FX400 relies on fluorescence-based detection, which can be affected by delayed loading, leading to fluorescence quenching and reduced detection values. This may cause positive specimens to go undetected if they do not meet the positive detection threshold.

Finally, differences in the ability to adsorb antimicrobial agents also play a role. After blood is drawn from septic patients, whether the pathogens grow and can be detected depends on the ability of the blood culture bottle’s contents to adsorb and neutralize antimicrobial agents in the sample. VIRTUO blood culture bottles enhance antimicrobial adsorption through three mechanisms: APB1 polymer beads adsorb certain antibiotics like ampicillin via ion exchange; APB2 beads adsorb larger molecules like vancomycin via van der Waals forces; and proprietary additives covalently bond with specific antibiotics such as carbapenems. In contrast, the FX400 blood bottles, developed earlier, do not effectively adsorb the wide range of modern antibiotics, which can lead to lower detection rates and an increased risk of missed infections. However, a potential limitation of the VIRTUO system is its relatively recent introduction to the market, meaning it may not be as widely available or implemented in lower-resource or smaller-scale settings as the more established BACTEC system. Additionally, one notable advantage of the BACTEC FX400 system is its broader use in many clinical settings due to its long-standing presence and widespread adoption in many laboratories around the world. The familiarity and established workflows associated with the BACTEC system can make it easier for laboratories to implement without the need for extensive retraining of personnel or major changes in infrastructure.

Compared to many current studies, this research offers several notable advantages and innovations. The comprehensive comparative analysis between the BacT/ALERT VIRTUO and BACTEC FX400 systems provides a detailed understanding of their relative efficiencies, which is more informative than studies that focused on a single system. For instance, a study conducted in Benin highlighted the high rates of antimicrobial resistance, particularly among Gram-negative bacteria, underscoring the critical need for improved blood culture detection methods to address antimicrobial resistance on a global scale. Similarly, prior comparative studies on BacT/ALERT and BACTEC systems have demonstrated that while both systems exhibit high detection rates, each system displays varying strengths in recovering Gram-positive and Gram-negative organisms. These findings align with our study, which further corroborates the differential performance of these systems in pathogen recovery (34, 35). One of the key innovations in this study is the use of dual-site blood collection. This approach minimizes the risk of false-negative results by ensuring a more comprehensive sample collection, thus improving the accuracy of detecting bloodstream infections. This method contrasts with previous studies that may have relied on single-site blood draws, potentially leading to underdiagnosis or incomplete pathogen recovery. Additionally, the present study’s large sample size increased the reliability and statistical power of the findings, allowing for more generalized and applicable conclusions in clinical settings. The large sample size represents an improvement over prior studies with smaller cohorts, providing more robust data for comparison and enabling a more precise evaluation of system performance across a diverse patient population. The dual-site blood collection methodology further enhanced the validity of the results, ensuring that observed differences were due to system performance rather than sample variability. Furthermore, conducting the study in a real-world clinical setting, specifically in Guangdong Provincial People’s Hospital, and involving a variety of hospital ward types, ensured that the findings are highly applicable to actual clinical practice. This study in China contributes valuable data from a new regional context, particularly in light of antimicrobial resistance trends and pathogen recovery. This pragmatic approach unequivocally ensured that the study’s recommendations were relevant and implementable in diverse healthcare environments (36).

Overall, this study stands out due to its comprehensive comparative analysis, large sample size, rigorous methodology, focus on early pathogen detection, detailed departmental performance analysis, and practical relevance. These innovations and advantages make it a valuable contribution to the field of clinical diagnostics and infectious disease management (37). The findings not only highlight the superior performance of the BacT/ALERT VIRTUO system but also provide actionable insights for improving blood culture diagnostics in diverse healthcare settings (38). By addressing both the technological and procedural aspects of blood culture diagnostics, the study provides a well-rounded perspective on how to enhance laboratory practices and patient care. However, this study also has certain limitations that should be acknowledged. One key limitation is the reliance on a single clinical setting, Guangdong Provincial People’s Hospital, which could limit the generalizability of the findings to other healthcare environments with different patient demographics, healthcare protocols, or resource availability. Additionally, the dual-site blood collection method, while innovative and beneficial in reducing false negatives, could introduce biases, as it may not be easily replicable in settings where resources or staffing are limited. Another limitation stems from potential variability in the experience levels of the healthcare professionals performing blood draws, which could impact sample quality and, by extension, diagnostic accuracy. Moreover, the study’s results may have been influenced by logistical challenges encountered during implementation, such as variations in ward practices or external factors affecting sample transport and handling.

Acknowledging these limitations provides a more nuanced interpretation of the results and suggests potential areas for future research. Despite these constraints, the comprehensive data and insights derived from this study can help guide clinical practice improvements and support the adoption of more effective diagnostic technologies in healthcare institutions (39, 40). The BacT/ALERT VIRTUO system, although requiring a higher upfront cost, could potentially offer cost savings over time due to reduced labor requirements, faster time-to-detection, and the associated clinical benefits that might lead to shorter hospital stays. To illustrate, the application of new microbiological technologies and the optimization of laboratory workflows have significantly shortened the time for microbiological reporting, thereby improving the timeliness of antibiotic selection and consequently reducing antibiotic usage costs for patients with bloodstream infections. Overall, the investment in faster reporting and workflow improvements is beneficial for treating patients with bloodstream infections and can yield substantial health economic benefits. In resource-poor settings, integrating automated blood culture systems could greatly improve diagnostic capabilities, yet requires adaptive strategies to overcome cost and infrastructure limitations.

Future research should focus on validating these findings in other clinical settings or populations. For example, we recommend introducing the dual-site blood collection method in clinical practice to reduce false-negative rates and optimize blood collection procedures, both in resource-rich and resource-limited settings. Further clinical trials in different regions or healthcare conditions, particularly in resource-limited environments, will help assess the effectiveness of this method in a broader context. Additionally, we suggest optimizing sample handling and processing procedures to minimize contamination rates, which will help improve diagnostic accuracy and reliability.

Moreover, this study raises several new questions that warrant exploration in future research. For instance, we intend to investigate whether the performance differences between the BacT/ALERT VIRTUO and BACTEC FX400 systems remain significant across various hospital departments or different healthcare systems. Additionally, we will explore innovative methods to reduce contamination in blood cultures, as enhancing the reliability of results is critical for improving patient outcomes. Furthermore, we recognize the importance of validating our findings in diverse clinical settings and populations to ensure the generalizability and sustainability of our research. Future studies could also examine the long-term impacts of improved detection times on patient management and outcomes, providing a more comprehensive understanding of how these systems can enhance clinical practice. Additionally, conducting a detailed analysis of performance variations depending on pathogen type would provide a more nuanced understanding of these systems' capabilities in detecting Gram-positive bacteria, Gram-negative bacteria, and fungi. This approach will refine their clinical applications and provide a more comprehensive perspective on how improved detection times might impact patient management and outcomes over the long term.

## SUPPLEMENTAL MATERIAL

Supplemental table spectrum.01850-24-s0001.docx Comparison of Cumulative TTD Between BacT/ALERT VIRTUO and BACTEC FX400 Systems.

ASM does not own the copyrights to Supplemental Material that may be linked to, or accessed through, an article. The authors have granted ASM a non-exclusive, world-wide license to publish the Supplemental Material files. Please contact the corresponding author directly for reuse.
