# Supplementary material for: Comparative evaluation of BacT/ALERT VIRTUO and BACTEC FX400 blood culture systems for the detection of bloodstream infections
Source: Microbiol Spectr. 2024 Nov 29;13(1):e01850-24. doi: 10.1128/spectrum.01850-24 (PMC11705859; doi:10.1128/spectrum.01850-24)
Supplement: Supplemental table — Comparison of Cumulative TTD Between BacT/ALERT VIRTUO and BACTEC FX400 Systems. [file spectrum.01850-24-s0001.docx]

**Supplementary table: Comparison of Cumulative TTD Between BacT/ALERT VIRTUO and BACTEC FX400 Systems**

| **Cumulative TTD** | **Positive detected** | **Group** | | **p-value**2 |
| --- | --- | --- | --- | --- |
|  |  | **FX400,N=185** | **VIRTUO,N=266** |  |
| **8h** | No | 176 (95.14%) | 211 (79.32%) | <0.001 |
|  | Yes | 9 (4.86%) | 55 (20.68%) |  |
| **16h** | No | 92 (49.73%) | 113 (42.48%) | 0.128 |
|  | Yes | 93 (50.27%) | 153 (57.52%) |  |
| **24h** | No | 53 (28.65%) | 57 (21.43%) | 0.079 |
|  | Yes | 132 (71.35%) | 209 (78.57%) |  |
| **32h** | No | 39 (21.08%) | 34 (12.78%) | 0.019 |
|  | Yes | 146 (78.92%) | 232 (87.22%) |  |
| **40h** | No | 28 (15.14%) | 16 (6.02%) | 0.001 |
|  | Yes | 157 (84.86%) | 250 (93.98%) |  |
| **48h** | No | 22(11.89%) | 7 (2.63%) | <0.001 |
|  | Yes | 163 (88.11%) | 259 (97.37%) |  |
| ^1^n(%) |  |  |  |  |
| ^2^Pearson's Chi-squared test |  |  |  |  |

This supplementary table provides a detailed comparison of cumulative TTD intervals between the BacT/ALERT VIRTUO and BACTEC FX400 systems. The table shows the number and percentage of positive cultures detected by each system at specified time points (8h, 16h, 24h, 32h, 40h, and 48h) and assesses statistical significance of detection rate differences at each interval using Pearson's Chi-squared test. These data highlight differences in the speed and efficiency of microorganism detection between the two systems.
